# Omicron breakthrough infections in vaccinated or previously infected hamsters

**DOI:** 10.1073/pnas.2308655120

**Published:** 2023-10-30

**Authors:** Jie Zhou, Ksenia Sukhova, Thomas P. Peacock, Paul F. McKay, Jonathan C. Brown, Rebecca Frise, Laury Baillon, Maya Moshe, Ruthiran Kugathasan, Robin J. Shattock, Wendy S. Barclay

**Affiliations:** ^a^Department of Infectious Disease, Imperial College London, London W2 1PG, United Kingdom

**Keywords:** SARS-CoV-2, hamster, transmission, reinfection, vaccine

## Abstract

The ongoing SARS-CoV-2 epidemic was marked by the repeated emergence and replacement of “variants” with genetic and phenotypic distance from the ancestral strains, the most recent examples being viruses of the Omicron lineage. Here, we describe a hamster direct contact exposure challenge model to assess protection against reinfection conferred by either vaccination or prior infection. We found that two doses of self-amplifying RNA vaccine based on the ancestral Spike ameliorated weight loss following Delta infection and decreased viral loads but had minimal effect on Omicron BA.1 infection. Prior vaccination followed by Delta or BA.1 breakthrough infections led to a high degree of cross-reactivity to all tested variants, suggesting that repeated exposure to antigenically distinct Spikes, via infection and/or vaccination drives a cross-reactive immune response. Prior infection with ancestral or Alpha variant was partially protective against BA.1 infection, whereas all animals previously infected with Delta and exposed to BA.1 became reinfected, although they shed less virus than BA.1-infected naive hamsters. Hamsters reinfected with BA.1 after prior Delta infection emitted infectious virus into the air, indicating that they could be responsible for onwards airborne transmission. We further tested whether prior infection with BA.1 protected from reinfection with Delta or later Omicron sublineages BA.2, BA.4, or BA.5. BA.1 was protective against BA.2 but not against Delta, BA.4, or BA.5 reinfection. These findings suggest that cohorts whose only immune experience of COVID-19 is Omicron BA.1 infection may be vulnerable to future circulation of reemerged Delta-like derivatives, as well as emerging Omicron sublineages.

## Significance

Since SARS-CoV-2 entered the human population, it has been constantly evolving, giving rise to new variants with enhanced transmissibility and/or antigenic escape from accumulating population immunity. We developed a hamster direct contact exposure challenge model to assess protection conferred by vaccination or prior infection against reinfection. Our results offer a significant contribution by validating neutralization assay-based antigenic cartography and assessing the potential of new variants to escape vaccine control or reinfect individuals with preexisting immunity. Our data underscore the importance of promptly updating vaccine immunogens based on detailed antigenic characterization validated by preclinical models to effectively control the spread of SARS-CoV-2 variants through vaccination strategies.

Omicron is the most recent SARS-CoV-2 Variant of Concern. Following its timely description by multiple laboratories in Africa in November 2021 (1), it spread rapidly displacing the previously circulating Delta variant around the world. BA.1 and BA.2 sublineages of Omicron circulated widely early in 2022, followed by BA.4 and BA.5 (2). More recently, multiple Omicron subvariants such as BQ.1 and XBB and further derivatives have increased rapidly in different areas (3). All Omicron variants carry a large number of mutations in their genome including over 30 coding changes in the Spike gene alone compared to the ancestral virus. This results in a considerable antigenic distance between Omicron Spike and that of other previous variants, especially Delta (4). Thus, it is not unexpected that antibodies induced after vaccination with the Spike of the early Wuhan strain, poorly neutralize the Omicron variants (5–13). The lack of cross-neutralization between Omicron and earlier variants likely accounts for the observed high transmission of Omicron lineage variants in populations that are heavily vaccinated and/or have a high rate of previous infection (14).

Vaccines utilizing encapsulated self-amplifying RNA (saRNA) possess distinct characteristics, including the ability to administer low doses and the ease of modifying the antigenic domain, enabling rapid vaccine formulation. Previously, we demonstrated that a saRNA encoding SARS-CoV-2 Spike protein encapsulated within a lipid nanoparticle (LNP) to be highly immunogenic in preclinical animal models (15, 16). Here, we use a hamster direct contact exposure challenge model of SARS-CoV-2 to illustrate Omicron BA.1 breakthrough infection after saRNA vaccine and also a high rate of reinfection by BA.1 in animals previously infected with Delta variant. Although the levels of BA.1 virus shedding from previously Delta-infected hamsters were much lower compared to naive hamsters, detection of infectious viruses in air from reinfected hamsters indicates the potential for onwards airborne transmission. We also find that hamsters previously infected with BA.1 were not protected against reinfection with Delta, BA.4, or BA.5 isolates but did not become infected after exposure to a BA.2 isolate. These studies offer an important contribution to calibrate in vitro neutralised assay-based antigenic cartography and to risk assess the potential for variants to escape vaccine control or to reinfect previously infected individuals. Taken together the results reinforce that there may be specific cohorts who are especially vulnerable to antigenically distant new variants, for example, children who have been less vaccinated than adults and often only with monovalent vaccines based on first-wave virus, or cohorts who have only experienced Omicron infections. Moreover, our findings imply that if we aspire to use vaccines to control circulation of SARS-CoV-2 variants, we will need a system for rapidly updating the immunogen based on detailed antigenic characterization validated with preclinical models.

## Results

### Omicron BA.1 Is Efficiently Transmitted to Hamsters Vaccinated with a Wuhan-Hu-1 Spike saRNA Vaccine.

We previously showed that immunisation with a self-amplifying RNA vaccine encoding the SARS-CoV-2 Wuhan Spike gene (saRNA-Spike) protected hamsters against weight loss following infection through exposure to cage mates infected with either a first-wave isolate or an Alpha isolate (15, 16). Although all exposed immunized hamsters in that study still became virus positive, weight loss and virus shedding were significantly reduced compared to that in a control group vaccinated with an irrelevant immunogen.

**Fig. 1.**
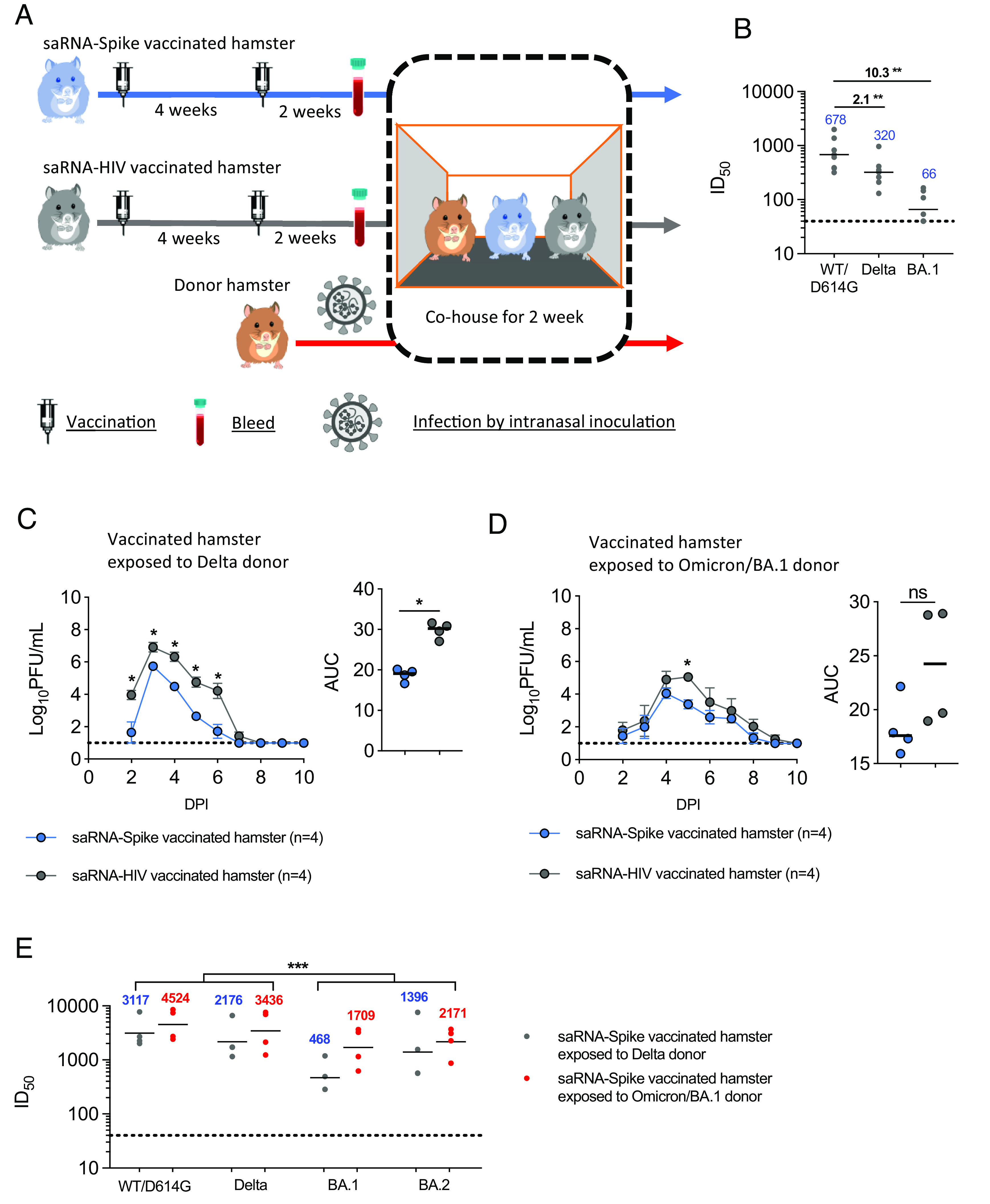
Delta and Omicron BA.1 infection of hamsters vaccinated with a self-amplifying Wuhan-Hu Spike RNA vaccine. (*A*) Experimental design. Two groups of hamsters were vaccinated with self-amplifying RNA (saRNA) Wuhan Spike or the saRNA-HIV vaccine. The vaccination schedule was a priming dose followed 4 wk later by a boost. Two weeks after the boost, the vaccinated hamsters were cohoused with donor hamsters which had been inoculated intranasally with 100 PFU Delta or BA.1 from 1 day post inoculation (DPI). Each cage housed one donor, one saRNA-Spike vaccinee, and one saRNA-HIV vaccinee hamster. (*B*) Pseudovirus neutralisation assays were performed using vaccinated hamster sera collected 2 wk after the boost (detection limit = 40, dotted line). Geometric means (blue) and fold changes (black) are shown. (*C* and *D*) The infectious virus shedding profile and area under the curve (AUC) in nasal wash samples of vaccinated hamsters exposed to Delta (n = 4 cages) (*C*) or BA.1 donors (n = 4 cages) (*D*). Nasal wash samples were collected daily and assessed by plaque assay (detection limit = 10 PFU/mL, dotted line). The symbols represent mean and SD in virus shedding curves, and median in AUC. (*E*) Pseudovirus neutralisation assays were performed using vaccinated hamster sera collected 2 wk after the exposure. Statistically significant differences were determined using the Mann–Whitney *U* test (*P < 0.05, **P < 0.01, and ***P < 0.001).

Here, we used the same protocol to test the efficacy of the saRNA-Spike vaccine against Delta and BA.1. Two groups of hamsters were immunized with either saRNA-Spike or a control vaccine encoding HIV gp120 (saRNA-HIV). The immunization regimen consisted of an initial priming dose, followed by a boosting dose 4 wk later (Fig. 1*A*). Two weeks postboost, serum samples were collected before challenge by the direct contact exposure route. Pseudovirus neutralization assays confirmed that hamsters vaccinated with saRNA-Spike had serum neutralizing titres against wild type (WT/D614G) [serum dilution that inhibits 50% infection (ID_50_) = 678, geometric mean] and showed twofold (*P* = 0.0078) and 10-fold (*P* = 0.0078) decrease in neutralizing activity against Delta and BA.1 respectively (Fig. 1*B*).

Unvaccinated donor hamsters were all productively infected intranasally with 100 PFU of either Delta or BA.1 and shed infectious virus in their nasal wash from day 1 post inoculation (1 DPI) as we reported previously (17). Vaccinated hamsters were cohoused with an infected donor from 1 DPI. Each cage thus housed one naive donor, one saRNA-Spike vaccinee and one control saRNA-HIV vaccinee hamster (Fig. 1*A*). Analysis of infectious virus (Fig. 1 *C* and *D*) and viral RNA (*SI Appendix*, Fig. S1 *A* and *B*) in nasal washes collected daily revealed that all contact hamsters became infected. However, both infectious viral load and viral RNA copies shed in nasal wash of saRNA-Spike vaccinated hamsters infected with Delta was significantly lower than that in the saRNA-HIV group on every day that virus was detected and in total [area under the curve (AUC)] (Fig. 1*C* and *SI Appendix*, Fig. S1*A*). Infectious viral load in nasal washes of saRNA-Spike vaccinated hamsters infected with BA.1 virus was less affected by vaccination and was only lower than in the saRNA-HIV vaccinated hamsters on 5 DPI (Fig. 1*D* and *SI Appendix*, Fig. S1*B*). Hamsters infected by Delta lost weight regardless of vaccine status; weight loss peaked at around 4.5% in the control group and at 3.2% in the saRNA-Spike vaccinated hamsters (*SI Appendix*, Fig. S1*C*). None of the hamsters infected with BA.1 in either the Spike or control vaccinated group lost weight, but they gained less weight compared to the mock-infected hamsters (*P* > 0.05) (*SI Appendix*, Fig. S1*D*). Thus, the reduction in neutralizing activity against BA.1 was associated with a compromised ability of the vaccine based on the first-wave Spike sequence (Wuhan) to reduce viral load.

### Sera from Vaccinated and Reinfected Hamsters Have Broader Cross Neutralizing Activity than Following Vaccination Alone.

We performed pseudovirus neutralization assays with sera collected 14 DPI of saRNA-Spike vaccinated hamsters. The postvaccine breakthrough infection sera from hamsters infected by BA.1 or Delta all had high neutralizing titres against WT/D614G, Delta, BA.1, and BA.2 (Fig. 1*E*), showing that vaccine breakthrough infection leads to a cross-reactive neutralizing response against both homologous and heterologous variants. Nonetheless, the neutralizing titres were lower (*P* = 0.0006) against BA.1 and BA.2 than against the WT/D614G and Delta variants, and sera from hamsters infected with Delta after saRNA-Spike vaccination were lowest against BA.1 (ID_50_ = 468).

### Hamsters Inoculated with Earlier Variants Are Reinfected Following Exposure to Omicron BA.1.

**Fig. 2.**
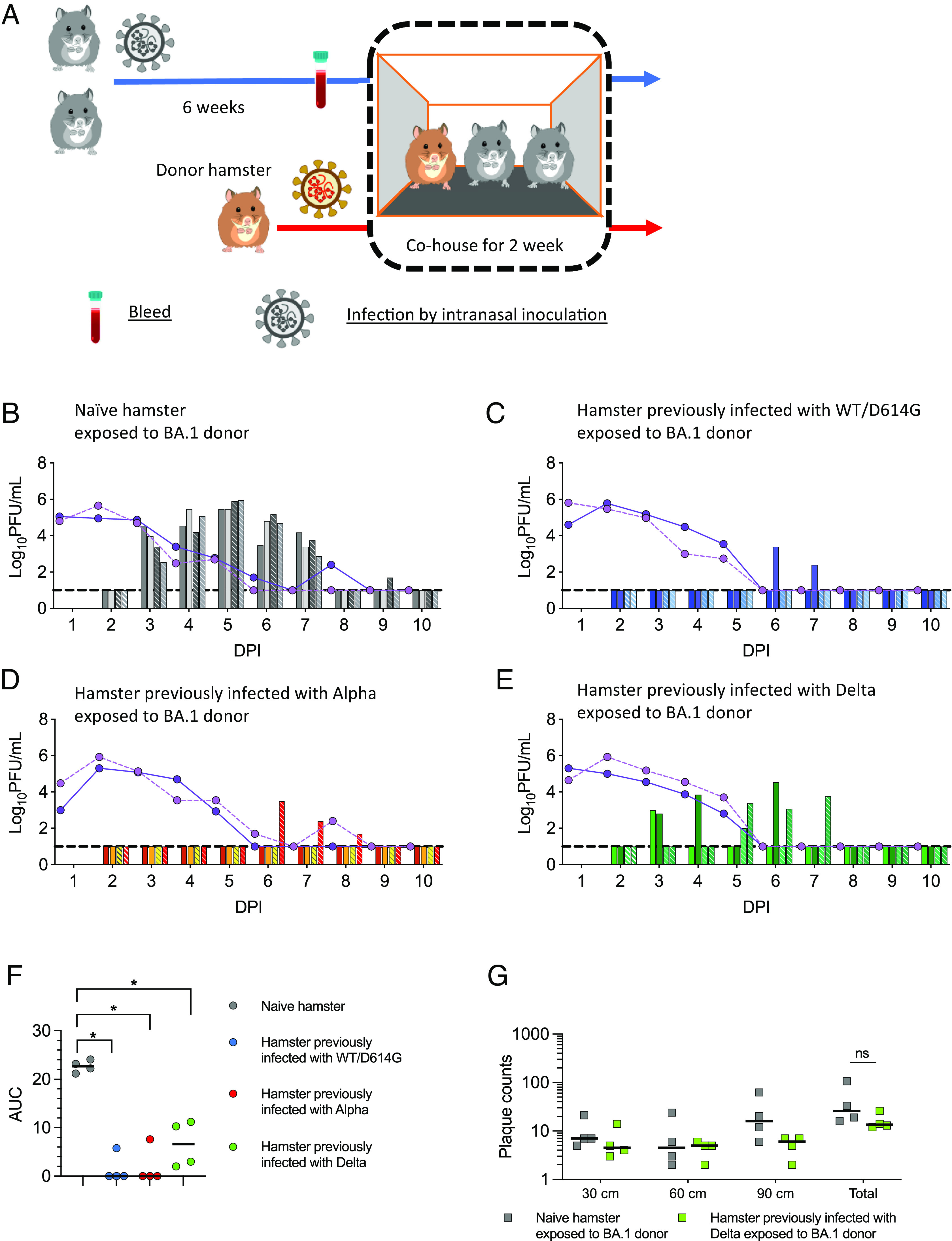
Reinfection of hamsters previously infected with earlier variants following direct contact exposure to Omicron BA.1. (*A*) Experimental design. Four groups of four hamsters each were inoculated intranasally with 100 PFU of either a wild-type isolate with D614G (WT/D614G), an Alpha isolate, a Delta isolate, or PBS. Six weeks later, two previously infected hamsters were cohoused with a donor hamster inoculated with 100 PFU of BA.1 from 1 day post inoculation (DPI). The direct contact transmission experiments were conducted in two cages (n = 2 biological replicates). (*B*–*E*) Virus-shedding profiles of donors (lines) and direct contact hamsters (bars) are shown. The hamsters in cage 1 are indicated by the solid line and unpatterned bars; the hamsters in cage 2 are indicated by the dotted line and patterned bars. (*B*) Naive hamster exposed to BA.1 donor. (*C*) Hamster previously infected with WT/D614G exposed to BA.1 donor. (*D*) Hamster previously infected with Alpha exposed to BA.1 donor. (*E*) Hamster previously infected with Delta exposed to BA.1 donor. Nasal wash samples were collected daily and assessed by plaque assay (detection limit = 10 PFU/mL, dash line). (*F*) AUC of infectious viral loads in direct contact hamsters. (*G*) Potential for onwards transmission determined by measuring infectious virus deposited from air at 30 cm, 60 cm, and 90 cm from the naive hamsters and the hamsters previously infected with Delta exposed to BA.1 donor on 3 DPI. Individual data points and median are shown (*F* and *G*). Statistically significant differences were determined using the Mann–Whitney *U* test (*P < 0.05, ns = not significant).

We next tested whether BA.1 would also reinfect hamsters that were previously infected with earlier variants (Fig. 2*A*). Groups of 4 hamsters each were infected via intranasal inoculation with 100 PFU of an early first wave wildtype isolate (WT/D614G), an Alpha isolate, a Delta isolate, or were mock-infected. All virus-inoculated hamsters shed virus in the nasal washes (*SI Appendix*, Fig. S2 *A–F*). Six weeks after the inoculation, serum samples were collected from the infected hamsters to test for the presence of neutralizing antibodies. We detected robust neutralizing titres against the homologous Spike within all groups, although due to limited serum volumes available we were not able to establish endpoint titres for all hamsters (*SI Appendix*, Fig. S2 *G*–*I*). Pseudovirus neutralization titres against BA.1 fell around or below the limit of detection in sera from hamsters infected with earlier variants. The previously infected or naive hamsters were then cohoused with donor hamsters infected intranasally with 100 PFU of BA.1 from 1 DPI. In total, eight donor hamsters were infected with BA.1 and randomly assigned for cohousing with the previously infected or mock-infected hamsters. The peak virus load and AUC were similar among the eight donor hamsters (*P* = 0.36, repeated measures ANOVA). All four naive hamsters acquired BA.1 infection from the cohoused donors and shed high levels of BA.1 virus in their nasal wash (Fig. 2*B* and *SI Appendix*, Fig. S2*J*). In contrast, only one of four hamsters previously infected with WT/D614G or Alpha shed infectious virus transiently after exposure to BA.1 donors (Fig. 2 *C* and *D*). Low levels of viral RNA were detected in all direct contact hamsters (*SI Appendix*, Fig. S2 *K* and *L*), suggesting virus deposited on respiratory tracts but failed to establish productive infection. Conversely, all four hamsters previously infected with Delta became reinfected upon exposure to BA.1 infected animals and shed infectious virus for several days postexposure (Fig. 2*E* and *SI Appendix*, Fig. S2*M*), albeit at lower titres than shed from naive hamsters infected with BA.1 (*P* = 0.0286) (Fig. 2*F*). None of the hamsters lost weight following infection with BA.1 regardless of previous SARS-CoV-2 infections, including the naive ones (*SI Appendix*, Fig. S3).

We also tested the potential of the reinfected animals for onwards transmission by measuring infectious virus exhaled in airborne droplets emitted by the infected animals using bespoke equipment (*SI Appendix*, Fig. S4) (18). At day 2 following the exposure to the inoculated donors (3 DPI), the four naive hamsters and the four previously Delta-infected hamsters were placed in a chamber from which air was passed over the surface of susceptible cultured cells. Following 10 min of exposure, the cells were removed and an overlay applied before 3-d incubation in order to observe plaques formed by infectious virus deposited on the cells. Cell culture plates were placed at three different distances, 30, 60, or 90 cm from the infected animal. All eight BA.1 infected hamsters emitted virus into the air, and overall plaque counts were not significantly different when comparing those from the naïve hamsters with those from the hamsters previously infected with Delta (*P* = 0.11) (Fig. 2*G*). The detection of infectious virus in the nasal washes and in the air emitted from the hamsters previously infected by Delta and reinfected by BA.1 suggests their potential to support onwards chain of transmission.

### Hamsters Previously Infected by Omicron BA.1 Are Reinfected When Exposed to Delta, BA.4, or BA.5 Variants, but Not BA.2.

Our data suggested that the hamster direct contact exposure model could be used to test the potential for SARS-CoV-2 variants to emerge because they could reinfect individuals previously infected with a preceding variant. Thus, the large antigenic distance of Omicron from Delta resulted in reinfection of previously Delta-infected hamsters by BA.1. Omicron sublineages have continued to emerge with increasing antigenic change during 2022 and 2023. We next challenged hamsters previously infected with BA.1 by exposure to the subsequent Omicron lineage viruses BA.2, BA.4, or BA.5 or to Delta to test for its potential to remerge, for example, from a chronically infected individual (19) or an animal reservoir (20).

**Fig. 3.**
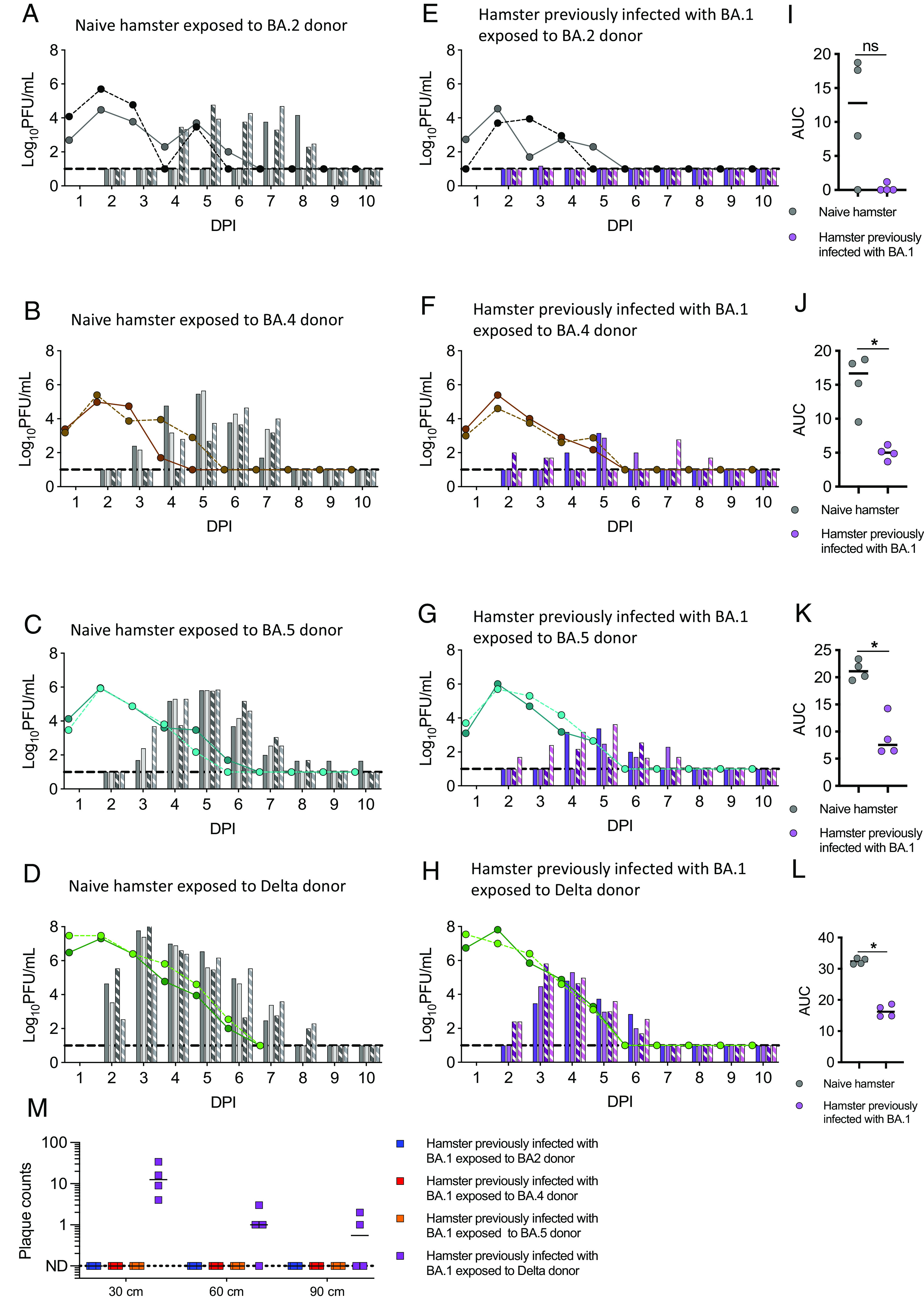
Reinfection of hamsters previously infected with Omicron BA.1 following direct contact exposure to BA.2, BA.4, BA.5, or Delta. Four groups of four hamsters each were inoculated intranasally by 100 PFU of BA.1. Six weeks later two previously inoculated hamsters were co-housed with a donor hamster inoculated with 100 PFU of either a BA.2, a BA.4, a BA.5 or a Delta isolate from 1 day post inoculation (DPI). (*A*–*H*) Virus-shedding profiles of donors (lines) and direct contact hamsters (bars) are shown. The hamsters in cage 1 are indicated by the solid line and unpatterned bars; the hamsters in cage 2 are indicated by the dotted line and patterned bars. (*A*–*D*) Naive hamster exposed to BA.2 donor (*A*), BA.4 donor (*B*), BA.5 donor (*C*), or Delta donor (*D*; two delta donors were killed on 7 DPI). (*E*–*H*) Hamsters previously infected with BA.1 exposed to BA.2 donor (*E*), BA.4 donor (*F*), BA.5 donor (*G*), or Delta donor (*H*). Nasal wash samples were collected daily and assessed by plaque assay (Detection limit = 10 PFU/mL, dash line). (*I*–*L*) AUC of infectious viral loads in the hamsters previously infected with BA.1 exposed to BA.2 donor (*I*), BA.4 donor (*J*), BA.5 donor (*K*), or Delta donor (*L*). (*M*) Potential for onwards transmission by measuring infectious virus deposited from air at 30 cm, 60 cm, and 90 cm from the hamsters previously infected with BA.1 exposed to BA.2, BA.4, BA.5, or Delta donor on 3 DPI. Individual data points and median are shown (*I*–*M*). Statistically significant differences were determined using the Mann–Whitney test (*P < 0.05, ns = not significant).

As before, the BA.1 infected hamsters were allowed to recover and convalesce for 6 wk following the initial infection. Sera collected at 6 wk postinoculation showed robust neutralizing titres against BA.1, but neutralizing activity against other variants was lower, with heterologous titres often at the limit of below detection in our assay (*SI Appendix*, Fig. S5 *A* and *B*). To test for susceptibility to reinfection, these hamsters were then cohoused with naive donor hamsters 1 d after the donors were inoculated with 100 PFU of BA.2, BA.4, BA.5 or Delta variants. All donors shed robust titres of infectious virus in their nasal wash (indicated by the lines in Fig. 3 *A–H*). However, we did note that virus titres in the nasal wash of the Delta inoculated donors were significantly higher than BA.2, BA.4, and BA.5 donors (*SI Appendix*, Fig. S5*C*). Three of the four naive hamsters exposed to the BA.2 donors were infected, and infectious virus shedding (indicated by the bars on Fig. 3*A*) became detectable in their nasal wash samples with a 1-d delay comparing to the kinetics of transmission measured for BA.1 (Fig. 3*A* compared with Fig. 2*B*). All four naive hamsters exposed to BA.4, BA.5, or Delta donors were infected and shed robust virus in nasal washes (Fig. 3 *B–D* and *SI Appendix*, Fig. S5 *E*–*G*). Infectious virus shedding became detectable 1 d after exposure for Delta, and 2 d after exposure for BA.4 and BA.5. Only one of the four hamsters previously infected with BA.1 was transiently reinfected by BA.2 (Fig. 3 *E* and *I* and *SI Appendix*, Fig. S5*G*). Titres of infectious virus were just above the detection limit and only evident on 3 DPI. All 4 previously BA.1 infected hamsters exposed to BA.4 or BA.5 inoculated donors were infected (Fig. 3 *F* and *G* and *SI Appendix*, Fig. S5 *H* and *I*) but shed much less virus compared to naive hamsters (Fig. 3 *J* and *K*). Direct contact hamsters infected with Omicron variants showed little weight loss (<5% maximum) regardless of previous infection history (*SI Appendix*, Fig. S6). In contrast to the situation above, where infection with an Omicron variant appeared to confer at least some protection against reinfection with a different Omicron subvariant, all four previously BA.1 infected hamsters were reinfected with Delta and robustly shed virus in nasal washes from 24 to 48 h after the exposure (Fig. 3*H* and *SI Appendix*, Fig. S5*J*). The virus shedding of these reinfected hamsters was lower than that observed in naive hamsters (Fig. 3 *D* and *L*), and they did not lose any weight (*SI Appendix*, Fig. S6*H*).

We also monitored infectious virus exhaled from the reinfected hamsters as a surrogate for the ability to support onwards airborne transmission. Following reinfection with BA.2, BA.4, or BA.5, none of the direct contact hamsters exhaled detectible virus-laden particles into the air. In contrast. infectious virus was detected from droplets emitted into the air 3 DPI by hamsters previously infected with BA.1 and reinfected by Delta (Fig. 3*M*). Overall, this shows that hamsters previously infected with BA.1 were reinfected with Delta, BA.4, or BA.5, and this was associated with antigenic distance measured by the decreased ability of antibodies in sera of previously infected animals to neutralize each variant (*SI Appendix*, Fig. S5 *A* and *B*). Reinfection by the virus with the greatest antigenic distance, Delta, resulted in high shedding of infectious virus from the nose and release of airborne virus with the potential to support chains of onwards transmission (Table 1).

**Table 1.** Breakthrough infections in previously vaccinated or infected hamsters

Self-amplifying RNA vaccinated	Direct contact exposed to virus	Infection confirmed by plaque assay	AUC (n = 4), (evaluated by plaque assay)	
saRNA-Spike	Delta	4/4 (100%)	15.5	Median of (16.5, 12.4, 15.1, 15.9)
saRNA-HIV	Delta	4/4 (100%)	27.8	Median of (28.6, 28.5, 24.1, 27.2)
saRNA-Spike	Omicron BA.1	4/4 (100%)	14.3	Median of (19.8, 14.9, 12.9, 13.7)
saRNA-HIV	Omicron BA.1	4/4 (100%)	21.8	Median of (27.0, 27.1, 16.7, 14.7)
Naïve or previously-infected				
Naive	Omicron BA.1	4/4 (100%)	22.7	Median of (22.2, 23.1, 24.1, 21.2)
WT/D614G	Omicron BA.1	1/4 (25%)	5.8	Single value
Alpha	Omicron BA.1	1/4 (25%)	5.9	Single value
Delta	Omicron BA.1	4/4 (100%)	6.6	Median of (3.0, 11.2, 2.0, 10.3)
Omicron BA.1	Delta	4/4 (100%)	16.20	Median of (14.9, 14.8, 17.6, 18.7)
Naive	Omicron BA.2	3/4 (75%)	17.6	Median of (8.0, 17.6, 18.8)
Omicron BA.1	Omicron BA.2	1/4 (25%)	1.0	Single value
Naive	Omicron BA.4	4/4 (100%)	16.7	Median of (18.1, 18.7, 9.5, 15.2)
Omicron BA.1	Omicron BA.4	4/4 (100%)	5.0	Median of (5.2, 4.9, 3.7, 6.2)
Naive	Omicron BA.5	4/4 (100%)	21.1	Median of (23.3, 20.2, 19.5, 22.0)
Omicron BA.1	Omicron BA.5	4/4 (100%)	7.5	Median of (8.6, 6.5, 6.4, 14.3)

## Discussion

The Syrian golden hamster has become the preclinical animal model of choice to assess vaccine effectiveness against SARS-CoV-2 variants, as well as the likelihood of reinfections (16, 21–28). Such information is critical for rational decisions around vaccine strain updates or other public health responses to the continuing emergence of new variants.

However, there are many different experimental protocols by which such assessments can be conducted and different outputs by which protection can be measured. Here, we employed a direct contact exposure challenge model, which introduces the challenge virus to naive, vaccinated or previously infected hamsters by cohousing them with donor animals already infected and shedding infectious virus. This more natural exposure route and dose may allow the model to be better calibrated with epidemiological observations in humans, and with the antigenic analyses that map the distance between variants using human serum and neutralization assays. In addition, we utilized a unique apparatus to measure virus emitted in breath from infected animals with or without prior immunity, to illustrate that in some breakthrough infections, despite reduced viral loads or amelioration of clinical signs, there is potential still for onwards transmission.

First, in our vaccine study, we employed a convenient saRNA vaccine encoding Wuhan Spike delivered in an LNP formulation. This is not a vaccine that is licensed for use in humans but is used here to induce antibodies and T cell responses against Wuhan Spike protein to mimic the immune experience of individuals who have received monovalent COVID-19 vaccines. Although in many parts of the world, vaccines have since been updated to bivalent formulations delivering both Wuhan and an Omicron spike, and more recently monovalent vaccines with Omicron immunogens, there are many vaccinees who have only received the first monovalent vaccines, for example, children and otherwise healthy adults in the United Kingdom (29). With the complex mixture of immune experience across populations, it is now difficult to find an immunization regimen that mimics human immunity. Here, we simply employed the Wuhan Spike saRNA vaccines to give the most basic immune response and serve as a baseline against which other vaccines or infections can be measured and correlated with immune responses.

We had previously developed our hamster direct contact challenge model to show that the Wuhan Spike saRNA vaccine conferred protection against weight loss and curtailed virus shedding of both homologous WT/D614G and heterologous Alpha variant (16). Similarly, in the present study, two doses of the same Wuhan Spike saRNA vaccine induced antibodies that correlated with decreased virus shedding upon Delta challenge. In contrast, the neutralizing activity induced by the vaccine against Omicron BA.1 was significantly reduced, and we did not observe a significant difference in BA.1 virus-shedding between the vaccinated group and the control group. Our results are in agreement with other studies using AZD1222 (ChAdOx1) or Moderna mRNA Wuhan Spike vaccines that also showed that vaccine-induced antibody titers were significantly lower against BA.1 compared to previous variants and virus shedding was similar to that of unvaccinated hamsters (21, 22).

Next, we used the hamster direct contact challenge model to investigate reinfection with BA.1 at 6 wk following their recovery from a previous infection with an earlier variant. We found that hamsters were reinfected after a first infection with WT/D614G or Alpha variant but only 1/4 in each group shed detectable infectious virus. In contrast, all the hamsters previously infected with Delta became productively reinfected with BA.1 and even emitted infectious virus in breath, implying potential for onwards transmission. This was despite that homologous titres to the first virus were similar or higher for WT/D614G or Alpha compared with Delta (*SI Appendix*, Fig. S2 *G*–*I*), and there was no difference in the titre of BA.1 virus shed from the donors to which each group was exposed (Fig. 2 *B–E*). The most obvious explanation for the more ready reinfection of Delta-experienced animals by BA.1 was the greater antigenic distance measured by the drop in neutralizing titre against BA.1. Importantly, several other groups have also reported a greater antigenic distance between Delta and Omicron than for other variants (30–32). The antigenic map created by Smith et al. showed that the WT/D614G and Alpha variants were more closely mapped to each other than either were to Delta, and the Omicron sublineages sit far apart and on the edge of the current map (33).

Our observation of limited BA.1 reinfection of hamsters previously infected with WT or Alpha variants differs slightly from those of Halfmann et al. who found all hamsters previously infected with WT/D614G were reinfected with BA.1 (22). This might be explained by the higher dose and direct route of challenge used in their study as well as the longer interval between the first and second infections. Nonetheless, in accordance with our findings, Halfmann et al. also observed reduced viral titres in nasal turbinates of prior-infected animals compared to naive animals. Shiwa-Sudo et al showed hamsters were reinfected with BA.1 after first infections with any of the prior variants but sustained lower viral RNA titres in nasal wash than in naive animals (23). In their study, hamsters previously infected with Delta were not more readily infected nor did they shed higher viral loads, in contrast to our findings. It is noteworthy that this group used an even higher direct inoculation challenge dose, so it is possible that this overrode the more subtle differences between variants that depend on antigenic distance. Several other groups have also assessed reinfection in the hamster model. Ryan et al. saw BA.1 reinfections of hamsters infected 50 d earlier with first-wave virus but puzzlingly also recorded reinfection with homologous virus at that time interval (26). Similarly, Plunkard et al. readily reinfected hamsters with BA.1 following first infections with a variety of different variants of concern (28). However they did not record virology outputs in their study but used weight loss as an indicator of infection making comparisons difficult.

Clearly, the outcomes of these experiments can depend on several parameters. We are interested to compare our results with epidemiological observations in humans. Using a Danish cohort with clinical metadata and sequence for both infections of individuals who had been sequentially infected, Burkholz et al. found that reinfections with Omicron after Delta were more frequent than those after Alpha (41% vs. 25%) (34). Additionally, until the emergence of Omicron, reinfections were relatively rare but the effectiveness of previous infection in preventing reinfection by Omicron was estimated to be 56.0% (35, 36). Thus, our challenge model where Omicron reinfections occur but to different degrees depending on the nature of the first infection might more accurately reflect the likelihood of reinfections in the community setting.

The final set of direct contact exposure challenges we carried out assessed reinfection of animals previously infected with Omicron BA.1 by later Omicron subvariants or by the preceding variant Delta. Here, we found that reinfection indicated by infectious virus shedding in nasal wash of all hamsters exposed to BA.4 and BA.5, but not BA.2. However, the BA.2 replicated poorly in donor animals and showed delayed transmission even in naive hamsters. Halfmann et al. had also noted that BA.2 sublineage was particularly unfit in hamsters (37). They also report hamsters were readily reinfected with XBB.1.5 after earlier infection with BA.1 (24). Omicron–Omicron reinfections were reported in epidemiological studies in Qatar (38) and are occurring over shorter time intervals (34) in line with this readily observed experimental reinfection of hamsters, suggesting that going forward these models can indeed be used to recapitulate and dissect the factors that are impacting the virus evolution. However, as SARS-CoV-2 continues to adapt to the human host, we need to remain cognizant that some variants may be attenuated in the hamster model and their transmission and fitness especially in immune animals might be underscored (37, 39).

On the other hand, Delta challenge resulted in high titre infectious virus shedding from hamsters previously infected with BA.1, including emissions of infectious virus in exhaled breath suggestive of onwards transmission. We cannot exclude that this robust reinfection was not explained by the high titres of Delta shed from the infected donors to which the BA.1 hamsters were exposed. Delta virus replicates very well in hamsters and Mohandas et al. also found hamsters infected with WT virus were readily reinfected with Delta (25). Nonetheless, the antigenic distance between Delta and BA.1 might also contribute to the high level of breakthrough infection of Delta after Omicron and raises some concern about the potential for future reemergence of historic Delta lineages seeded into immunocompromised people or animal hosts (19, 20).

Reassuringly, the breadth of antibody-neutralizing response is increased after reinfection with heterologous virus. We observed that Omicron BA.1 breakthrough infection in saRNA-Spike vaccinated hamsters generated potent and broad neutralizing activity against other variants of concern, including Omicron BA.1 and BA.2. This suggests that repeated exposure to antigenically distinct Spike proteins, through infection and/or vaccination, induces a more cross-reactive immune response. These findings align with reports indicating that Omicron BA.1 breakthrough infection leads to potent antibody cross-reaction in vaccinated populations (40–42) and hold implications for the development of COVID-19 vaccine strategies.

The limitations of a study such as this are inevitably the small number of animals making it difficult to validate those cases where there appears to be protection following prior infection. However, the high reinfection rates observed in hamsters previously infected with different variants provide confidence in the results obtained. A second limitation is the difference in susceptibility of hamsters to different variants noted above that could impact the interpretation of results. Third, we only studied short-term immunity (6 wk) conferred by natural infection or vaccination confers, understanding the duration and longevity of the protection against reinfection with new variants is of utmost importance. Interestingly, even with the short time interval, we still observed 100% reinfection of Delta (primary infection) -BA.1 (reinfection), BA.1-BA.4, BA.1-BA.5, and BA.1-Delta reinfections. Conducting additional studies with varying time intervals between primary infection and reinfection would allow us to better understand the dynamics of immune protection and identify the impact of waning immunity.

In conclusion, our study emphasizes the utility of the hamster direct exposure challenge model to test vaccine efficacy and potential reinfection with emerging SARS-CoV-2 variants. Such in vivo experiments can help calibrate measurements of the neutralizing antibody response to give indications of when we expect antigenic mismatch to result in significant levels of breakthrough infections. Our findings provide insights into the continued circulation of Omicron sublineage variants, and this information could contribute to evidence-based public health policies.

## Materials and Methods

### Biosafety and Ethics Statement.

All work performed was approved by the local genetic manipulation (GM) safety committee of Imperial College London, St. Mary’s Campus (centre number GM77), and the Health and Safety Executive of the United Kingdom, under reference CBA1.77.20.1. Animal research was carried out under a United Kingdom Home Office License, P48DAD9B4.

### Cells and Viruses.

Human embryonic kidney cells (293 T; ATCC; ATCC CRL-11268) were maintained in Dulbecco’s modified Eagle’s medium (DMEM; Gibco), 10% fetal calf serum (FCS), 1% non-essential amino acids (Gibco), and 100 U/mL penicillin-streptomycin (P/S; Gibco). Stably transduced ACE2-expressing 293 T cells were produced as previously described (43) and maintained with the addition of 1 μg/mL puromycin to growth medium. African green monkey kidney (VeroE6) cells expressing human angiotensin-converting enzyme 2 (ACE2) and transmembrane protease serine 2 precursor (TMPRSS2) (VAT) were kindly provided by MRC-University of Glasgow Centre for Virus Research, Glasgow (44). The cells were maintained in DMEM, 10% FCS, 1 mg/mL Geneticin (Gibco), 0.2 mg/mL Hygromycin B (Invitrogen). All viral stocks used in this study were grown in the VAT cells: WT/D614G (hCoV-19/England/IC19/2020, B.1.13, EPI_ISL_475572), Alpha (hCoV-19/England/205080610/2020, B.1.1.7, EPI_ISL_723001), Delta (hCoV-19/England/SHEF-10E8F3B/2021, B.1.617.2, EPI_ISL_1731019), BA.1 (hCoV-19/England/M21021166/2022, BA.1), BA.2 (hCoV-19/England/IC243335/2022, BA.2), BA.4 (hCoV-19/England/clin64/2022, BA.4). and BA.5 (hCoV-19/England/NWLP_53/2022, BA.5).

### Plaque Assays.

Nasal wash samples were serially diluted in DMEM and added to the VAT cell monolayers for 1 h at 37 °C. Inoculum was then removed and cells were overlayed with DMEM containing 0.2% bovine serum albumin (Gibco), 0.16% NaHCO_3_ (Gibco), 10 mM 4-(2-hydroxyethyl)-1-piperazineethanesulfonic acid (HEPES) (Invitrogen), 2 mM L-Glutamine (Gibco), 100 U/mL P/S and 0.6% Avicel (Gibco). Plates were incubated at 37 °C, 5% CO_2_ for 3 d. The overlay was then removed, and monolayers were stained with 0.05% crystal violet solution for 1 h at room temperature. Plates were washed with tap water then dried and virus plaques were counted. The lower limit of detection of the assay was 10 plaque forming units per mL (PFU/mL).

### SARS-CoV-2 E/ORF1a Gene qRT-PCR.

Virus genomes were quantified by Envelop (E) or Open reading frame 1a (ORF1a) gene qRT-PCR as previously described (45, 46). Viral RNA was extracted from supernatants of hamster nasal wash samples using the QIAsymphony DSP Virus/Pathogen Mini Kit on the QIAsymphony instrument (Qiagen). The qRT-PCR was then performed using the AgPath RT-PCR (Life Technologies) kit on a QuantStudio™ 7 Flex Real-Time PCR System. For absolutely quantification, a standard curve was generated using dilutions viral RNA of known copy number. Gene copies per mL of original virus supernatant were then calculated using this standard curve. The lower limit of detection of the qRT-PCR was 1,200 copies per mL.

### Hamster Transmission Studies.

Hamster transmission studies were performed in a containment level 3 laboratory, using ISO Rat900 Individually Ventilated Cages (IVC) (Tecniplast, Italy). Outbred male Syrian Hamsters (4 to 6 wk old), weighing 80 to 130 g were used. In the vaccine study, hamsters were immunized twice, 4 wk apart with a saRNA vaccine encoding either SARS-CoV-2 Spike protein or a control vaccine encoding HIV gp120 protein, intramuscularly in 100 μL. Donor hamsters were intranasally inoculated with 50 μL of 100 PFU of each virus while lightly anaesthetized with isoflurane. The vaccinated hamsters were introduced into the same cage with an infected donor day 1 after inoculation. Each cage thus housed one donor, one saRNA-Spike vaccinee and one control saRNA-HIV gp120 vaccinee animal. In the reinfection studies, naive hamsters were intranasally inoculated with 100 PFU of virus. Six weeks later, two previously infected hamsters were introduced into the same cage with an infected donor from day 1 post inoculation (1 DPI). Each cage thus housed one donor and two direct contact hamsters from 1 DPI to 14 DPI. All animals were nasal washed daily by instilling 400 μL of phosphate-buffered saline (PBS) into the nostrils, the expectorate was collected into a 50-mL falcon tube. Hamsters were observed and weighed daily postinfection.

The potential for hamsters infected with SARS-CoV-2 to transmit onwards was assessed using a set of equipment which detects infectious virus exhaled from infected animals as described previously (18). Airflow of 4.5 L/min was introduced using the bias flow pump via three ports into a 10 cm (height) × 9 cm (diameter) hamster chamber (1.5 L/min into each port). Sentinel cell (VAT) culture plates were placed at three different distances, 30 cm, 60 cm, or 90 cm from the infected animal source.

### Pseudovirus Neutralization Assays.

SARS-CoV-2 Spike-bearing lentiviral pseudotypes were generated as described previously (43, 47). Pseudovirus neutralization assays were performed by incubating serial dilutions of heat-inactivated convalescent antisera with a set amount of pseudovirus. Antisera/pseudovirus mix was then incubated at 37 °C for 1 h then overlayed into 96-well plates of 293 T-ACE2 cells. Forty-eight hours later, cells were lysed with reporter lysis buffer (Promega) and assays were read on a FLUOstar Omega plate reader (BMF Labtech) using the Luciferase Assay System (Promega).

### Statistical Analysis.

Statistical analysis was performed using Graphpad Prism (Version 10.0.1). Group comparisons were tested using Mann–Whitney *U* test for unpaired groups and Wilcoxon matched-pairs signed rank test was used for paired groups. ANOVA was used to compare virus shedding profile of donor hamsters. For all tests, a value of *P* < 0.05 was considered significant.

## Supplementary Material

Appendix 01 (PDF)

## Data Availability

All study data are included in the article and/or *SI Appendix*.
