# Supplementary material for: Omicron breakthrough infections in vaccinated or previously infected hamsters
Source: Proc Natl Acad Sci U S A. 2023 Oct 30;120(45):e2308655120. doi: 10.1073/pnas.2308655120 (PMC10636328; doi:10.1073/pnas.2308655120)
Supplement: Supplementary file 1 — Appendix 01 (PDF) [file pnas.2308655120.sapp.pdf]

PNAS

Supporting Information for

Omicron breakthrough infections in vaccinated or previously infected hamsters

Authors: Jie Zhou[1 †], Ksenia Sukhova[1†], Thomas P. Peacock[1†], Paul F. McKay[1], Jonathan C. Brown[1], Rebecca Frise, Laury Baillon, Maya Moshe[1], Ruthiran Kugathasan[1], Robin J. Shattock[1], Wendy S. Barclay[1]*

Wendy S. Barclay
Email: w.barclay@imperial.ac.uk

This PDF file includes:

Figures S1 to S6

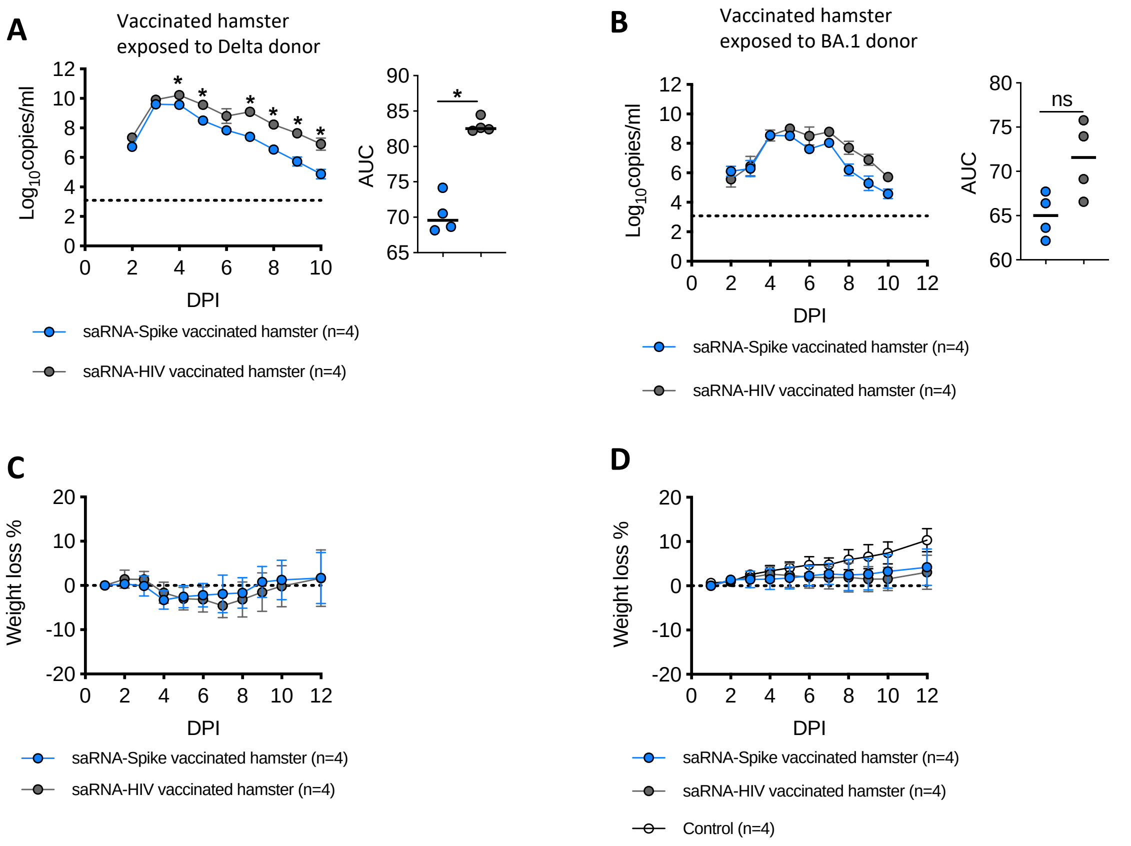

Fig. S1. Delta and Omicron BA.1 infection of hamsters vaccinated with a self-amplifying Wuhan-Hu-1 Spike RNA vaccine. (A, B) Virus shedding profile and area under curve (AUC) of saRNA vaccinated hamsters exposed to either delta donor (A) or BA.1 donor (B). Nasal wash samples were collected daily and assessed by RT-qPCR. The lower detection limit is 1200 copies/mL (dotted lines). (C, D) Body weight change of saRNA vaccinated hamsters exposed to either delta donor (C) or BA.1 donor (D). The symbols represent mean and S.D. in virus shedding profiles and weight loss change, and median in AUC. Statistically significant differences were determined using Mann-Whitney test (* $p < 0.05$).

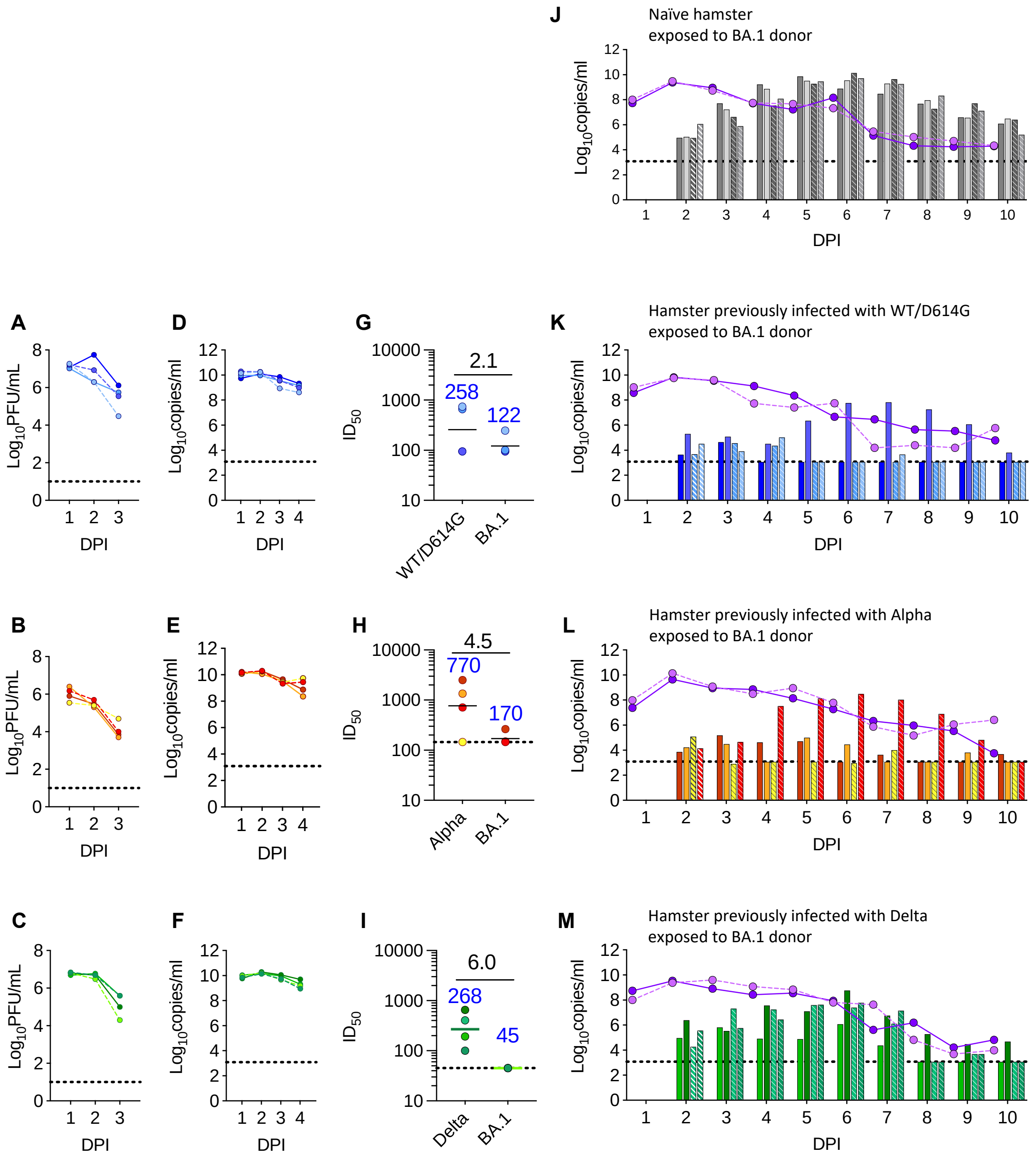

Fig. S2. Reinfection of hamsters previously infected with earlier variants following direct contact exposure to Omicron BA.1. (A-F) Groups of 4 hamsters each were inoculated with 100 PFU of either a WT/D614G (A,D), Alpha (B, E), or Delta (C, F) isolate. Nasal wash samples were collected daily from 1dpi to 3dpi and titrated by plaque assay (A-C) and qPCR (D-F). The detection limit of plaque assay and qPCR is 10 PFU/mL and 1200 copies/mL respectively (dotted line). (G-I) Pseudovirus neutralisation assays against homologous strain and BA.1 using sera collected from hamsters previously infected with WT/D614G (G), Alpha (H) or Delta isolate (I). Individual data points and median are shown (G -I). (J-M) Virus shedding profiles of donor hamsters (lines) and direct contact hamsters (bars) are shown. (J) Naïve hamster exposed to BA.1 donor. (L) Hamsters previously infected with WT/D614G exposed to BA.1 donor. (L) Hamsters previously infected with Alpha exposed to BA.1 donor. (M) Hamsters previously

infected with Delta exposed to BA.1 donor. Nasal wash samples were collected daily and assessed by qPCR. Colour-matched dots and bars represent results from the same hamster.

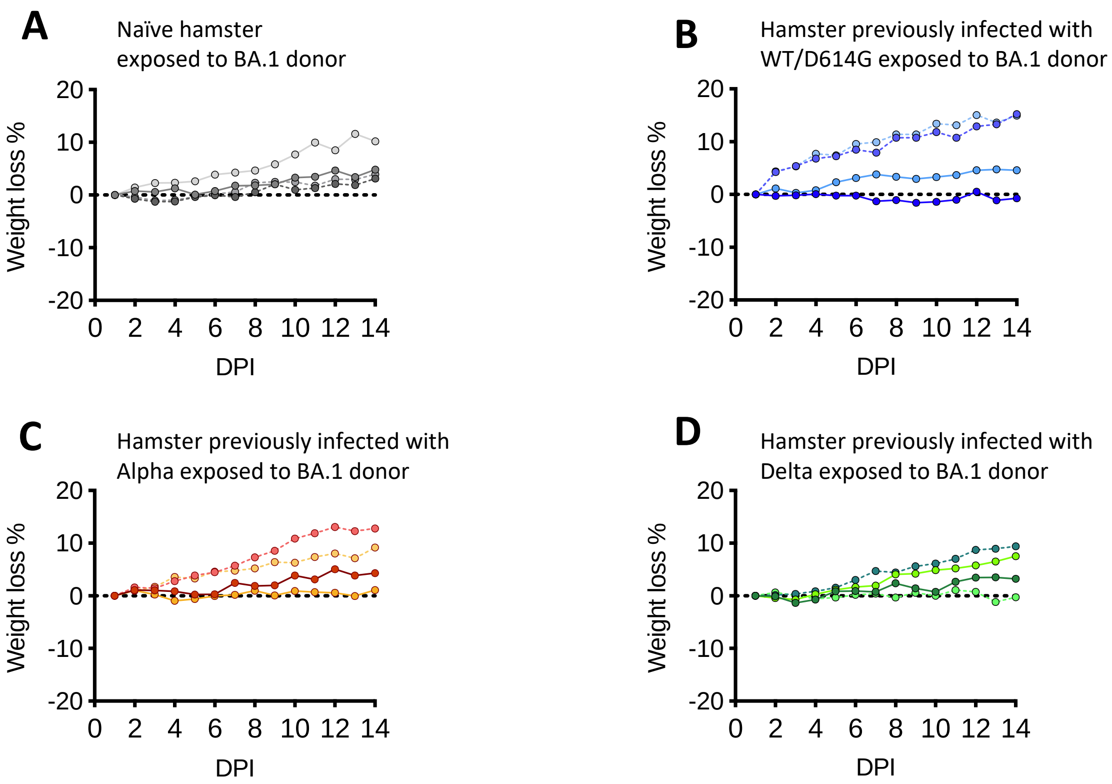

Fig. S3. Weight loss change of direct contact hamster exposed to Omicron BA.1 donor. Body weight change of hamsters was monitor daily. (A) Naïve hamster exposed to BA.1 donor. (B) Hamster previously infected with WT/D614G exposed to BA.1 donor. (C) Hamster previously infected with Alpha exposed to BA.1 donor. (D) Hamster previously infected with Delta exposed to BA.1 donor.

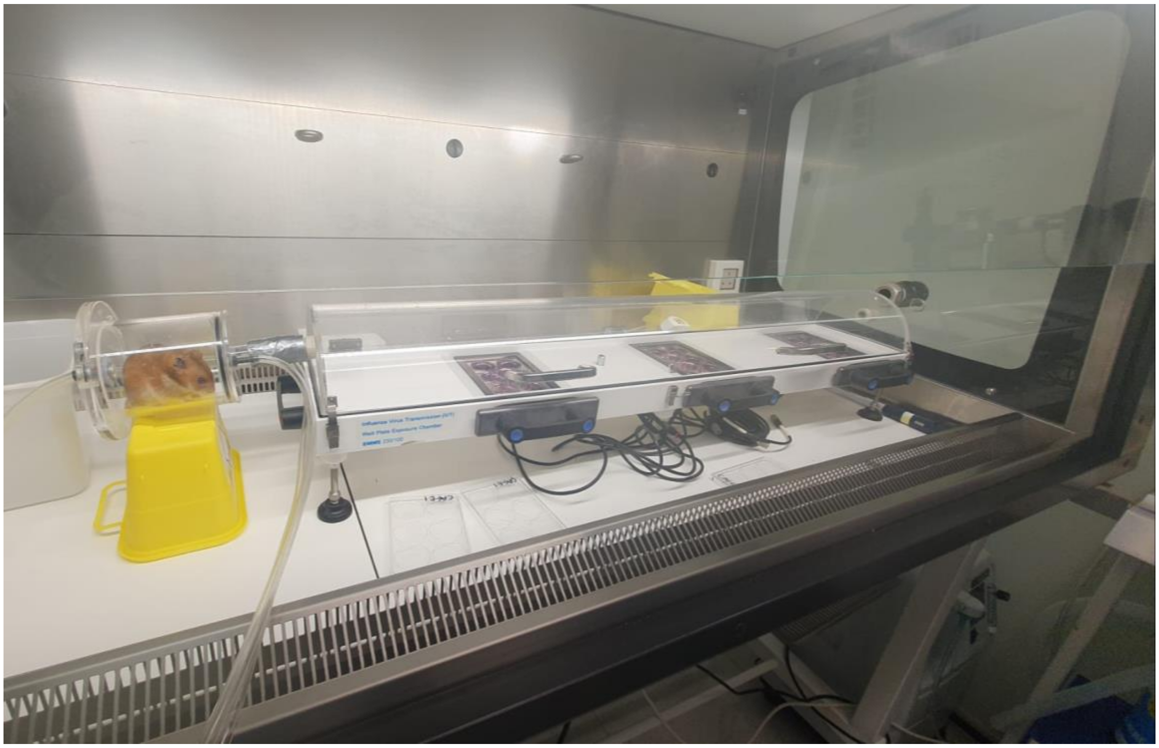

Fig. S4. Assessing infectious virus exhaled from infected hamsters. Airflow (4.5 Liter/minute) is generated using a bias flow pump, which connects to a hamster chamber (10cm x 9cm, long x diameter). The chamber is connected to a half cylindrical clear acrylic 100cm (length) x 18cm (width) x 9cm (height) exposure tunnel containing cell culture plates situated 30cm, 60cm and 90cm from the animal. The hamsters were sampled for 10 minutes.

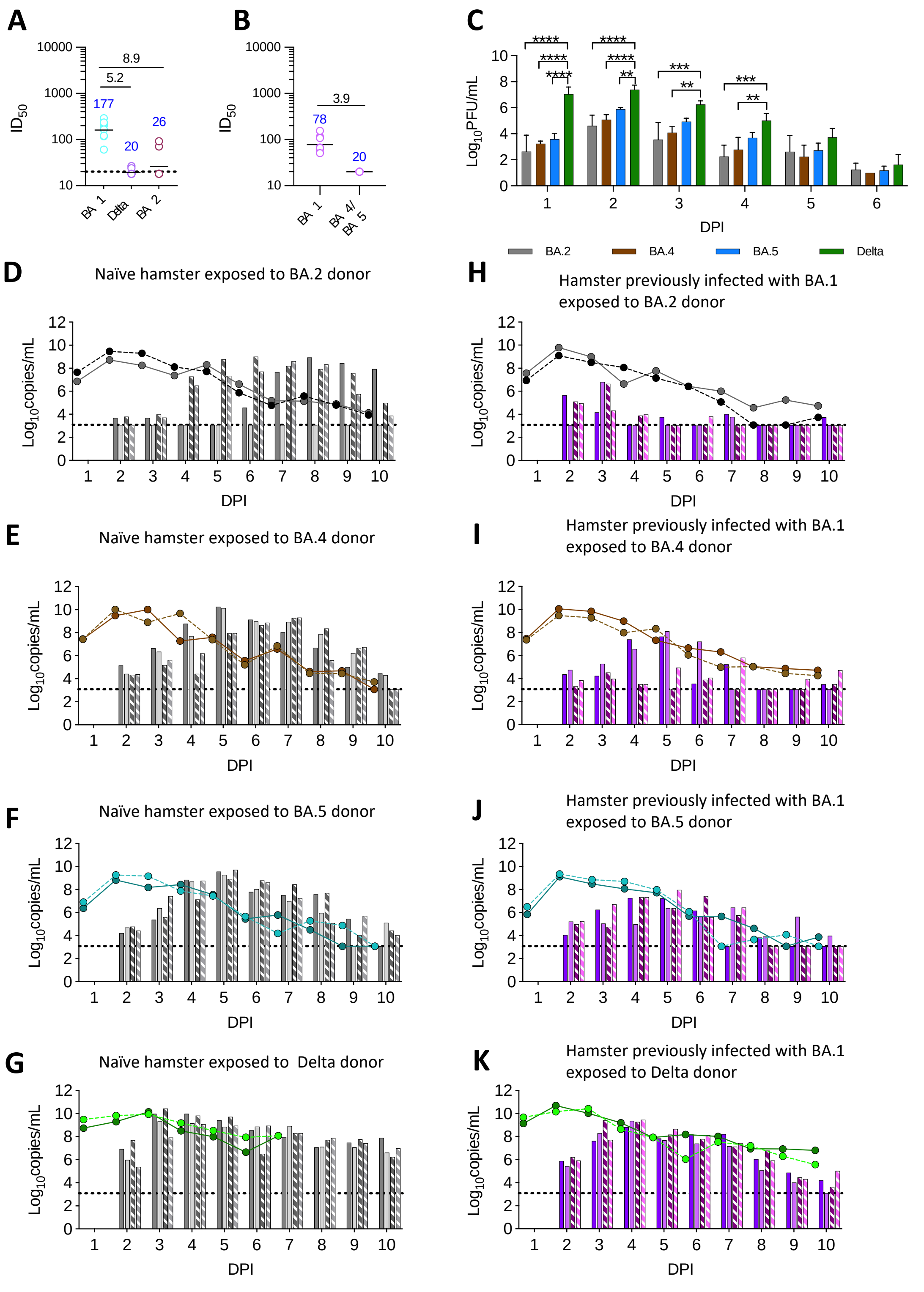

Fig. S5. Reinfection of hamsters previously infected with Omicron BA.1 following direct contact exposure to BA.2, BA.4, BA.5 or Delta. (A, B) Pseudovirus neutralisation assays using sera collected six weeks later after BA.1 inoculation. Neutralisation activity in sera collected from previously BA.1 infected hamsters exposed to Delta or BA.2 donor (n = 8) (A) or exposed to BA.4

or BA.5 donor (n = 8) (B). Geometric means (blue) and fold changes (black) are shown. (C) Virus-shedding profiles of donor hamsters inoculated with Delta, BA.2, BA.4 or BA.5. The mean and S.D. are shown. Statistically significant differences were determined using one-way ANOVA, followed by Dunnett's multiple comparison test (**** $p < 0.0001$, *** $p < 0.001$, and ** $p < 0.01$). (D-K) Virus-shedding profiles of donor hamsters (lines) and direct contact hamsters (bars) are shown. (D-G) Naïve hamster exposed to BA.2 donor (D), BA.4 donor (E), BA.5 donor (F) or Delta donor (G; two delta donors were euthanised on 7DPI). (H-K) Hamster previously infected with BA.1 exposed to BA.2 donor (H), BA.4 donor (I), BA.5 donor (J), or Delta donor (K). Nasal wash samples were collected daily and titrated by qPCR. The detection limit for qPCR is 1200 copies/mL (dotted line).

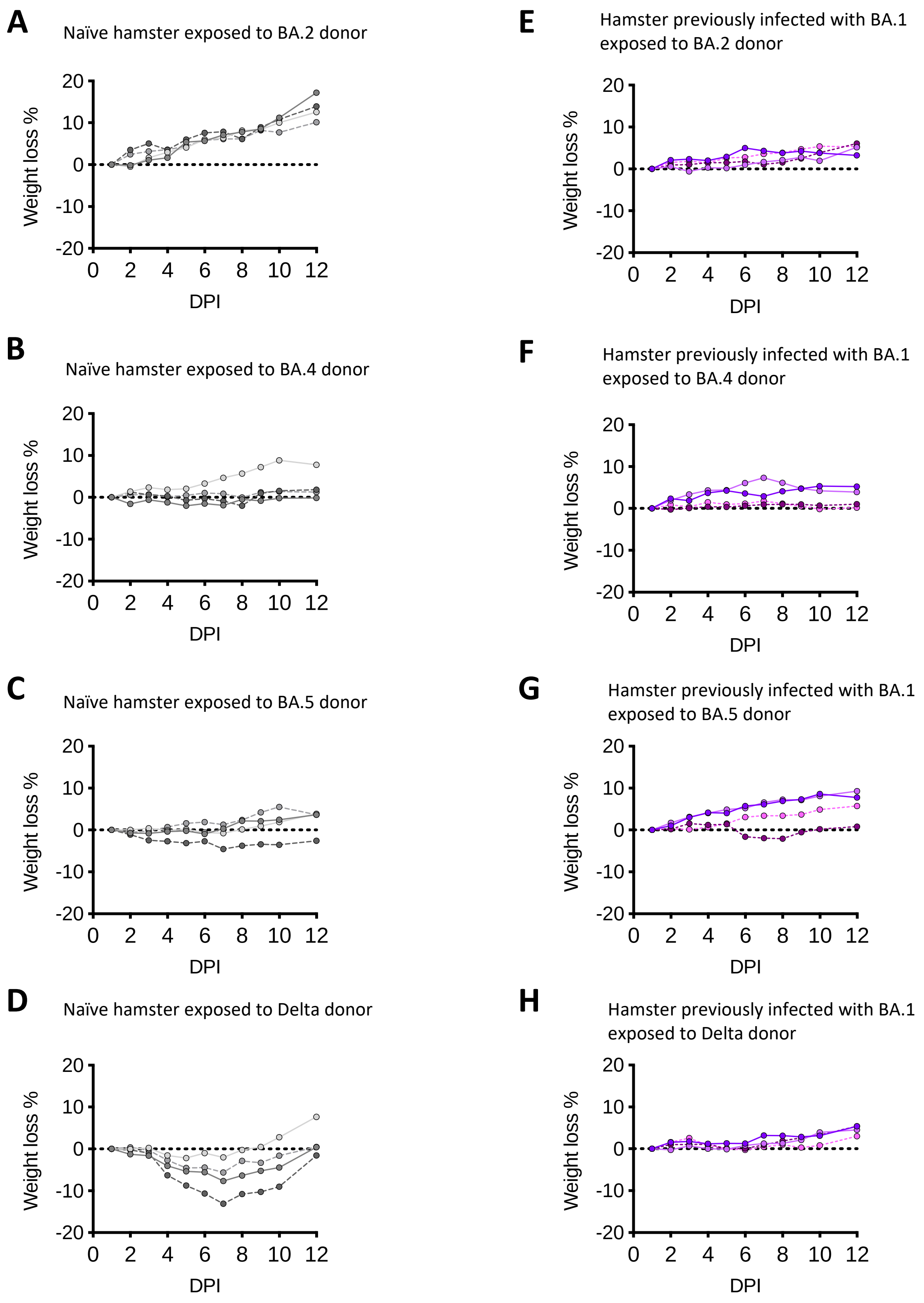

Fig. S6. Weight loss change of direct contact hamsters following exposure. Body weight change was monitored daily. (A-C) Naive hamster exposed to BA.2 donor (A), BA.4 donor (B), BA.5

donor (C) or Delta donor. (D-G) Hamster previously infected with BA.1 exposed to BA.2 donor (E), BA.4 donor (F), BA.5 donor (G), or Delta donor (H).
